# PReS-FINAL-2284: SLE and complement deficiencies: a French multicentric retrospective study

**DOI:** 10.1186/1546-0096-11-S2-P274

**Published:** 2013-12-05

**Authors:** B Roland-Gosselin, E Allain-Launay, E Plouvier, L Mouthon, AL Fauchais, JC Lega, H Remeaux, P Cochat, M Desjonquères, M -H Said-Menthon, B Bader-Meunier, A Belot

**Affiliations:** 1 Hopital Femme Mère Enfant, Bron, France; 2 Service de néphrologie pédiatrique, Hopital Mère Enfant, Nantes, France; 3 Service de Pédiatrie, Hôpital Saint-Jacques, Besançon, France; 4 Service de Médecine Interne, Cochin, Paris, France; 5 Service de Médecine Interne, CHU, Limoge, France; 6 Service de Médecine Interne, CH Lyon Sud, Lyon, France; 7 Service de Néphrologie, Rhumatologie & Dermatologie pédiatriques, Hopital Femme Mère Enfant, Bron, France; 8 Service de Pédiatrie, Hopitaux du Léman, Thonon-les-Bains, France; 9 Unité d'Immunologie-Hématologie et Rhumatologie Pédiatriques, Necker-Enfants Malades, Paris, France

## Introduction

Systemic lupus erythematosus (SLE) is a multifactorial disease. Rare causes of monogenic SLE have been described, including the complement deficiencies.

## Objectives

Our objectives were to collect clinical data and outcome of SLE patients associated to complement deficiency in a multicenter retrospective study.

## Methods

We conducted a retrospective study within the French paediatric rheumatology society (SOFREMIP) in 2012-2013.to identify patients, with a confirmed deficiency of complement fraction.

## Results

Ten cases of SLE with complement deficiency were identified: 2 C1 deficiencies, 2 C2 deficiencies and 6 partial C4 deficiencies. The sex ratio (M/F) is 0/10. The disease onset occurred in childhood in 8 patients with 6 before the age of 10. The first symptoms were cutaneous in 7 children, articular for 2 children and psychiatric for 1 patient. All patients were positive for antinuclear antibodies whereas only half of them were positive for anti-dsDNA antibodies. Anti-Ro (SS-A) antibodies were strongly positive in 8 patients. Anti-phospholipidantibodies were presentin 6 patients. Over time, 5 patients developed a severe disease associated to renal failure (n = 2) or neurolupus (n = 3). Associated autoimmune diseases were found in 4 patients: hypothyroidism (n = 1), autoimmune hepatitis (n = 2), Sjögren syndrome (n = 1). Two children with C2 and C4 deficiency had severe bacterial infections.

## Conclusion

Cutaneous or joint manifestations are the most common symptoms but life-threatening complications can occur in the context of C1 deficiency. Anti-SSA antibodies were frequent while anti-DNA are only found in half of the cases. Genetic characterization of complement deficiencies remains challenging. Next generation sequencing may be helpful to better diagnose these monogenic forms of lupus.

## Disclosure of interest

None declared.

